# Multisite Infection with *Mycobacterium abscessus* after Replacement of Breast Implants and Gluteal Lipofilling

**DOI:** 10.1155/2015/361340

**Published:** 2015-03-29

**Authors:** Eva Rüegg, Alexandre Cheretakis, Ali Modarressi, Stephan Harbarth, Brigitte Pittet-Cuénod

**Affiliations:** ^1^Division of Plastic, Reconstructive and Aesthetic Surgery, Department of Surgery, Faculty of Medicine of the University of Geneva, Geneva University Hospitals (HUG), Rue Gabrielle Perret-Gentil 4, 1205 Geneva, Switzerland; ^2^13 Avenue Krieg, 1208 Geneva, Switzerland; ^3^Division of Infectious Diseases, Department of Internal Medicine, Faculty of Medicine of the University of Geneva, Rue Gabrielle Perret-Gentil 4, 1205 Geneva, Switzerland

## Abstract

*Introduction*. Medical tourism for aesthetic surgery is popular. Nontuberculous mycobacteria (NTM) occasionally cause surgical-site infections. As NTM grow in biofilms, implantations of foreign bodies are at risk. Due to late manifestation, infections occur when patients are back home, where they must be managed properly. *Case Report*. A 39-year-old healthy female was referred for acute infection of the right gluteal area. Five months before, she had breast implants replacement, abdominal liposuction, and gluteal lipofilling in Mexico. Three months postoperatively, implants were removed for NTM-infection in Switzerland. Adequate antibiotic treatment was stopped after seven days for drug-related hepatitis. At entrance, gluteal puncture for bacterial analysis was performed. MRI showed large subcutaneous collection. Debridement under general anaesthesia was followed by open wound management. Total antibiotic treatment was 20 weeks. *Methods*. Bacterial analysis of periprosthetic and gluteal liquids included Gram-stain plus acid-fast stain, and aerobic, anaerobic and mycobacterial cultures.  *Results*. In periprosthetic fluid, *Mycobacterium abscessus, Propionibacterium*, and *Staphylococcus epidermidis* were identified. The same *M. abscessus* strain was found gluteally. The gluteal wound healed within six weeks. At ten months' follow-up, gluteal asymmetry persists for deep scarring. *Conclusion*. This case presents major complications of multisite aesthetic surgery. Surgical-site infections in context of medical tourism need appropriate bacteriological investigations, considering potential NTM-infections.

## 1. Background

### 1.1. Medical Tourism

Medical tourism for aesthetic surgery has gained popularity [1]. There are various motivations for seeking healthcare abroad and one of the most important is cost [2, 3]. Another advantage is discretion. Patients may be attracted by the idea of combining beach holidays with aesthetic surgery [3]. This combination allows hiding aesthetic surgery behind an adventurous holiday. Traditional touristic destinations are therefore attractive for such purpose [4]. Some immigrants prefer going back to their homeland to get the more familiar way of healthcare [5, 6] and to combine family visits with surgery [7]. Common procedures are liposuction, abdominoplasty, and breast implants.

Medical tourists are at risk for infections [8]. The prevalence of healthcare-related infections in developing countries is substantially higher than in Europe and the United States and is even double the rates for surgical-site infections [9].

Aesthetic surgery seems to be hit rarely but regularly by surgical-site infections due to nontuberculous mycobacteria (NTM). Different reports of clusters of infections caused by NTM following breast surgery, abdominoplasty, and liposuction have been reported from South America (Venezuela and Dominican Republic [10–12]) and North America [13], affecting both local patients and medical tourists.

### 1.2. Breast Implant Infections

Infections after breast augmentation with implants occur in 2-3% of aesthetic procedures [14–17]. Generally, neither the type of implant nor the localisation of the incision seems to have a significant influence on the rate of infection. Acute infections occur within the first month after implantation [18]. They usually present clinically with fever and an erythematous painful breast. In most cases, there is a periprosthetic fluid collection, which may be punctured under ultrasonic vision to confirm diagnosis and identify pathogens. Concerned bacteria are most frequently* Staphylococcus* (S.)* aureus* and* S. epidermidis*, Streptococci, Enterobacteriaceae, and* Pseudomonas* spp. [18]. Sources of infection may be contracted perioperatively from patient's skin, contamination of surgical instruments, or endogenous flora of the breast. The breast gland is in contact with the environment through its ducts. Cultures of nipple swabs and of occlusive perioperative dressings showed a bacterial contamination rate of 35%, mostly with species of the normal skin flora such as coagulase-negative staphylococci such as* S. epidermidis* [19]. Bacterial analysis of surgically removed breast specimen was positive for coagulase-negative staphylococci in 42 to 53%, and 30 to 52% of the cultures showed no bacterial growth [20]. Other microorganisms identified are anaerobic* Propionibacterium acnes*, diphtheroids and lactobacilli,* Bacillus* spp., and beta-haemolytic streptococci.

Late infections, occurring after more than one month postoperatively, are observed more rarely. In a large study cohort of 10,941 patients operated on by 265 surgeons, a rate of late infection of only 0.8% was reported [16]. They are usually due to hematogenic spreading during bacteraemia of invasive procedures, typically dental infection surgery [16]. Secondary spreading to breast implants is reported from different sources with their typical microorganisms [18, 21].

Periprosthetic NTM-infections in breast augmentation are rare but regularly reported [22–25]. NTM-infections typically occur later than acute infections, between 3 and 8 weeks postoperatively, but with similar symptoms [26]. Due to their biological particularities, NTM cannot be found in routine bacteriologic analysis and are not covered by conventional perioperative antibiotic prophylaxis.

Very few reports on NTM-infections of lipofilling sites exist so far [10, 27, 28] and to the best of our knowledge multiple-sites infection occurring after a combined operation has not been published before.

## 2. Case Report

The patient was a 39-year-old female with a body mass index (BMI) of 18.6 kg/m^2^ in good general health living in Switzerland. She was referred to us with an erythematous painful induration of the right gluteal area.

**Figure 1:**
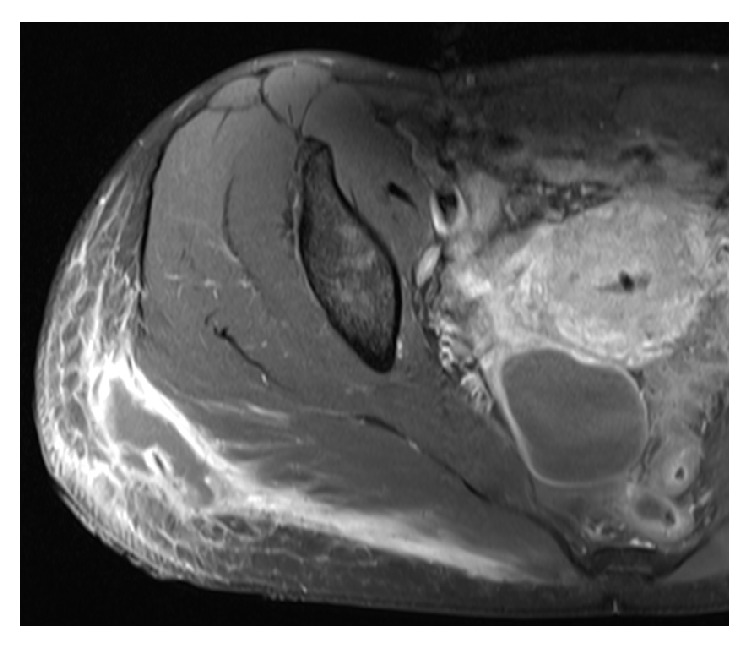
MRI shows T1 phase with gadolinium contrast, showing a small superficial gluteal collection joining large deep subcutaneous collections in contact with the muscular fascia, associated with diffuse infiltration of the neighbouring subcutaneous tissue and the gluteus maximus muscles.

**Figure 2:**
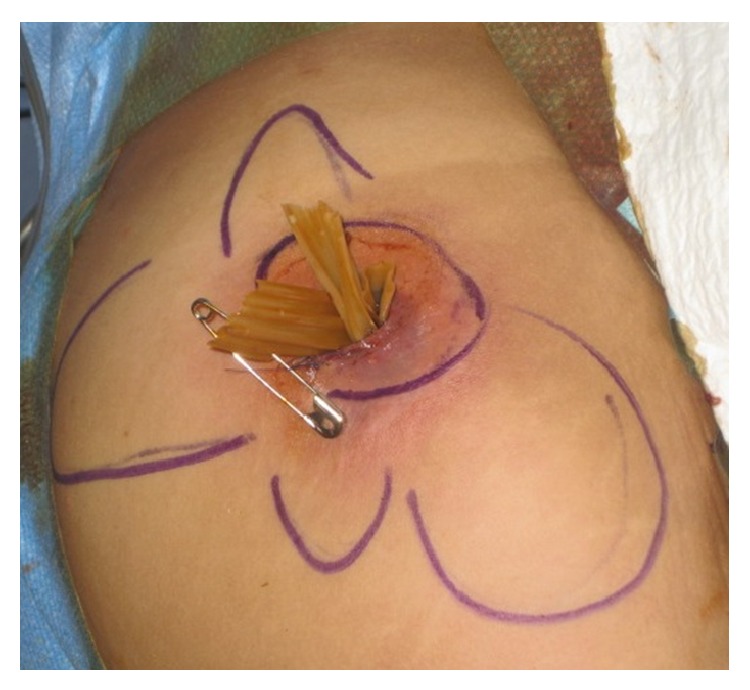
Intraoperative view after debridement of the gluteal collection and placement of drains in undermined areas (drawn in blue).

Five months before, she had bilateral breast implants replacement via submammary approach with a left periareolar mastopexy, abdominal liposuction, and gluteal lipofilling in a private hospital in Mexico. Since the procedure, she felt her left breast swollen, but without pain or erythema. Two months after surgery, she observed a progressive painful enlargement of the left submammary scar. Magnetic resonance imaging (MRI) showed nothing specific. The patient consulted a private practice for left submammary scar disunion and oozing 13 weeks postoperatively. She had no fever, but small amount of periprosthetic fluid and inflammatory signs around the submammary scar were visible on ultrasounds. Bilateral implants removal was performed the next day in a private hospital in Switzerland for clinical suspicion of implants infection. Intraoperatively, the left periprosthetic capsule was covered with a gelatinous film, which was removed and sent for bacteriologic and histopathologic analysis, asking for specific mycobacteria culture. The wound was washed out with a solution containing gentamicin. An antibiotic therapy with amoxicillin/clavulanic acid and ciprofloxacin was introduced. The bacteriological examination found* Mycobacterium abscessus *subspec.* massiliense*,* Propionibacterium *spp., and* Staphylococcus epidermidis* and the intravenous antibiotic treatment was changed for imipenem, amikacin, and clarithromycin for seven days, administered in an inpatient setting. Local signs of infection rapidly disappeared, and the antibiotic treatment had to be interrupted after one week due to drug-related hepatitis. Formal antibiotic susceptibility testing of the NTM strain was performed, respecting the Swiss version of the European Committee on Antimicrobial Susceptibility Testing guidelines, and revealed sensibility to amikacin and clarithromycin, intermediate sensibility to linezolid, imipenem, and cefoxitin, and resistance to tobramycin, moxifloxacin, ciprofloxacin, and doxycycline. Simultaneously with breast implant infection, the patient felt some pain and the presence of a small subcutaneous node in the right gluteal region. She did not mention this observation to the surgeon performing the breast implant removal, who was even not aware of the liposuction and lipofilling procedures she had three months before. The pain in the right gluteal area was released during the period of the antibiotic treatment for the breast implant infection. However, pain and inflammation came back and worsened in the following four weeks with erythema and induration after the premature stop of antibiotic treatment. Because the patient was abroad at that time, a treatment with linezolid was introduced and rapidly changed to clarithromycin. Nevertheless, the gluteal lesion progressed with an increased swelling and erythema. At the arrival to our hospital, she had no fever; the leucocyte count was normal and C-reactive protein was at 64 mg/L. An ultrasonographically guided puncture of the gluteal abscess was performed for bacterial analysis. In respect of pathogens found on implant removal, the empiric antibiotic therapy of clarithromycin was enlarged with tigecycline, linezolid, and amikacin. MRI showed a large subcutaneous collection of 4 × 7 × 10 cm in contact with the muscular fascia, associated with a diffuse infiltration of the neighbouring subcutaneous tissue, the gluteus maximus muscle, and the presence of an ipsilateral inflammation of the sacroiliac joint (Figure 1). The patient was taken to the operation room and the abscess was drained under general anaesthesia. A large amount of necrotic fat was removed and all deep cavities were debrided using large curette. The size of the undermined area was much larger than shown on the MRI, reaching the midline. Drains were placed in every direction of the undermining. Controlled open wound healing was achieved washing the wound along the drains with diluted povidone-iodine solution (Betadine, Mundipharma Medical Company) twice a day for four days. Drains were progressively removed and daily dressings were replaced by intermittent negative-pressure wound-therapy at postoperative day eight for four weeks (Figure 2). Six weeks after surgical debridement, the wound was healed.

Ziehl-stain performed on the puncture of the right gluteal abscess revealed the presence of the same strain found in the breast analysis of* Mycobacterium abscessus *subsp.* massiliense*. Formal antibiotic susceptibility testing of the NTM strain showed again sensibility to amikacin and clarithromycin as well as intermediate sensibility to linezolid and cefoxitin; furthermore, it showed resistance to tobramycin, moxifloxacin, ciprofloxacin, imipenem, and doxycycline. Tigecycline and amikacin were stopped after 12 days on request of the patient due to nausea and linezolid was stopped after 30 days for cutaneous rash and drug-induced hepatitis. Monotherapy with clarithromycin was maintained for additional 17 days. It was then enlarged with moxifloxacin for a small contralateral gluteal collection found on the control-MRI along with the persistence of the asymptomatic inflammation of the right sacroiliac joint. A sonographically guided puncture and biopsy of the subcutaneous portion of the contralateral gluteal collection revealed the absence of mycobacteria. A fluorine-18 fluorodeoxyglucose positron emission tomography combined with cervicothoracoabdominal computerized tomography confirmed the absence of further inflammatory zones than the healed right gluteal area, and moxifloxacin was stopped after a total therapy of 6 weeks. Monotherapy with clarithromycin was pursued for a total duration of 20 weeks.

**Figure 3:**
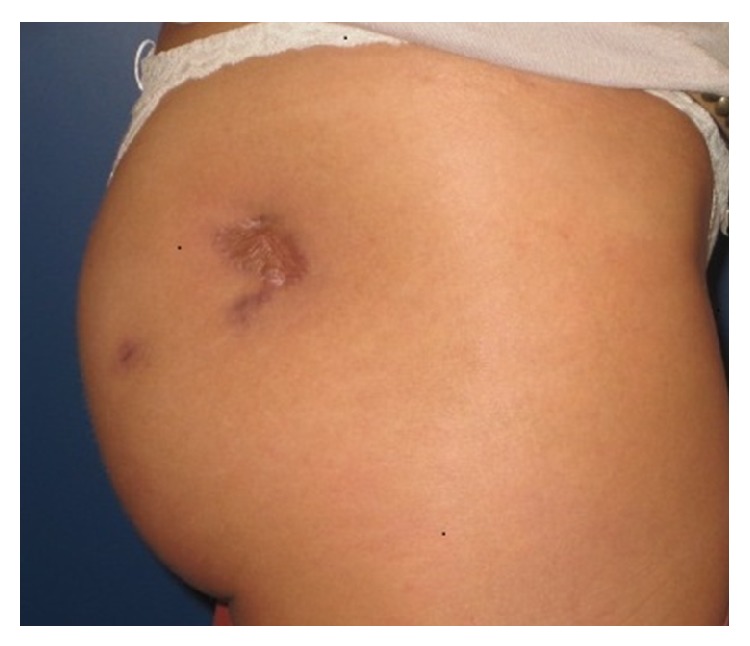
At six months' follow-up, a hyperpigmented scar of a three-centimeter diameter was present in the middle of a depressed area of about seven centimetres in the right gluteal region. The scar is not hidden in a conventional swimming suit.

The clinical outcome ten months postoperatively and five months after termination of the antibiotics showed no sign of infection recurrence. A hyperpigmented scar of a three-centimeter diameter was present in the middle of a depressed area of about seven centimetres in the right gluteal area. There is obvious gluteal asymmetry compared to the contralateral side (Figure 3).

## 3. Discussion

NTM are ubiquitous in nature and are found in biofilms in aqueous systems [29, 30]. They are relatively resistant to standard disinfectants such as chlorine, organomercurials, and alkaline glutaraldehydes [31, 32]. Possible sources of surgical-site infections may occur due to the use of tap water for cleaning surgical instruments followed by inadequate disinfectants and sterilization procedure [33]. Operations with implantation of a foreign body are, therefore, especially at risk. There are many reports of NTM-surgical-site infections with different types of implants. Besides breast implants [22–25], other implants have been concerned such as periorbital implants, orthopaedic implants, contraceptive implants, and penile implants [34–40]. A NTM-contaminated skin marking solution has also been reported to be responsible for a series of postoperative infections after various aesthetic procedures performed by a single surgeon [41].

Since NTM have a slower reproductive circle than the typical pathogens responsible for acute infections, the clinical signs appear later. Additionally, their diagnosis is often delayed for two reasons. First, they cannot be found with conventional bacteriological analysis; only acid-fast stains (Ziehl-Neelsen) and acid-fast cultures can reveal the presence of mycobacteria. Second, cultures need be kept for at least 14 days. During the waiting time for the bacteriology results, routinely administered antibiotic treatments like betalactam agents are not effective against NTM. For faster recognition of NTM, Lowenstein-Jensen media or the BACTEC MB900 automated instrumentation is useful [42]. Another diagnostic tool is the histopathological examination to search for granuloma and acid-fast organisms [42].

In this case report, the source of infection could not be clearly identified. As the same strain of* Mycobacterium abscessus *subspec.* massiliense* was found in both examinations of breast and gluteal specimens, contamination during the surgical procedure is highly likely. Surprisingly, the breast implant and the grafted fat of only one side were infected, and the liposuction abdominal area was free from infection.

The pathogen is known for its ability to form biofilms on inert material, which is represented in this case by the breast implants. Necrotic fat grafts could equally act as a foreign body and be a good base for biofilm formation. In the presented case, only the right gluteal area became infected. Injection technique is known to be important for the fat graft survival. Only small amounts of fat should be injected in multiple channels, in order to assure the access to well-perfused surrounding tissue [43]. If large quantities are injected into the same place, the centre of the injected fat will not be adequately reperfused and undergoes necrosis. In the reported case, the injected fat on the contralateral healthy side presented as a small visible collection without clinical signs of infection. The infected side showed a much larger collection suspect for local overfilling of the grafted fat.

Supposing that contamination of the surgical instruments was the source, one would expect all operated sites to get infected. The fact that the abdominal donor site of the fat graft, the right breast, and left buttock were not concerned by infection reveals that if foreign body presents a specific risk for NTM-infection, it is not the only factor. It underlines that cautious surgical technique avoiding necrosis or collection is of particular importance in presence of NTM-contamination. Vascularized tissue in an immuno-competent patient is certainly less vulnerable to infection.

Interestingly, the surgeon in Mexico who performed the first operation was informed of the infection, but he did not report any other cases with the same type of infection.

Nowadays, in the time of tourism for aesthetic surgery, patients from Western Europe travel to South America, North Africa, or Eastern Europe [44]. Local follow-up is then obviously very short, and performing surgeons are often not aware of late results and occurring complications. For instance, several outbreaks of NTM surgical-site infection have only been revealed by concerted action of different public health authorities in several states in the United States [12]. In 2007, a survey of the British Association of Plastic, Reconstructive and Aesthetic Surgeons with a response rate of 62% showed that 37% of the respondents had seen patients who presented with complications or concerns related to cosmetic procedures abroad [45].

The case presented here is an example of a major complication of a multisite aesthetic procedure on a healthy, beautiful young woman. She needed two additional operations under general anaesthesia and prolonged antibiotic treatment for more than five months. The patient developed twice a drug-related hepatitis due to the antibiotic treatment and had other side effects as nausea and cutaneous rash.

There are minor sequelae from the removal of the infected breast implants regarding loss of breast projection. The second complication of the infected gluteal lipofilling needed open wound healing, which demanded regular dressings for six weeks. Five months after the end of the antibiotic treatment, there is no sign of infection. The enlarged scar is still present in a depressed and hyperpigmented gluteal area which is not completely hidden in a swimming suit.

This paper aims to clear up the importance of a correct technique of lipofilling without overfilling in order to limit the formation of necrotic tissue. Furthermore, potential additional risk of combined aesthetic procedures must be clearly addressed with the patient preoperatively. Surgical-site infections are known to be significantly higher in developing countries [9, 46, 47]. These elements should be taken into consideration by candidates for such a kind of procedure. Possible scenarios of multisite infections as described in this case must be thought of, if one site gets infected. When late infections occur after more than one month postoperatively, acid-fast staining and mycobacterial cultures over at least 14 days should be performed.

## 4. Conclusion

This case presents a major complication of a multisite aesthetic surgery performed in a developing country. Inadequate injection technique may result in fat necrosis, increasing the risk for NTM-infection. Surgical-site infections in the context of medical tourism have to be managed with appropriate bacteriological investigations, having in mind a potential NTM-infection.
